# Letter from New Castle, Ky.

**Published:** 1853-10

**Authors:** C. P. Culver


New Castle, Ky., *Jan.* 15*th*, 1853.

*Dr. Taylor*—Dear Sir: I have been using, or rather experimenting, with the following chemical compound, and with the most happy effects; so far as to give instant relief to pain in the teeth or jaws:

1 oz. Hartshorn;
1 oz. Chloroform;

2 dr. Oil Cloves;
2 dr. Nitric Acid;
2 dr. Ex. Camphor;
2 dr. Ex. Nutgall.

I have been observing, as minutely as practicable, the peculiar effects this compound has had upon the teeth; and, from the experience I have had in its use, and my observation of its effects, I am led to the conclusion, that it may be used with safety as a sedative—and decidedly with benefit—in those cases where the operator is not permitted to perform the necessary operations—provided care and attention be given to prevent discoloration of the teeth, which will arise from its use.

I would like to have your opinion as to its peculiar chemical effects upon the teeth, as well as the propriety of its use.

Yours, &c., C. P. Culver.

[All these articles, in some shape or other, have been recommended for tooth-ache, and also more or less for obtunding the sensibility of the dentine previous to plugging. The only article of the compound to which we should especially object is the nitric acid. This, we apprehend, would act injuriously on the teeth.—Ed.]

